# Consumo de Frutose Altera Aminas Biogênicas Associadas a Fatores de Risco Cardiovasculares

**DOI:** 10.36660/abc.20220770

**Published:** 2023-06-07

**Authors:** Fabiane Valentini Francisqueti-Ferron, Matheus Antônio Filiol Belin, Thiago Luiz Novaga Palacio, Artur Junio Togneri Ferron, Jéssica Leite Garcia, Juliana Silva Siqueira, Erika Tiemi Nakandakare-Maia, Taynara Aparecida Vieira, Hugo Tadashi Kano, Fernando Moreto, Giuseppina Pace Pereira Lima, Camila Renata Corrêa, Igor Otavio Minatel

**Affiliations:** 1 Universidade Estadual Paulista Júlio de Mesquita Filho Faculdade de Medicina Botucatu SP Brasil Universidade Estadual Paulista Júlio de Mesquita Filho Câmpus de Botucatu Faculdade de Medicina, Botucatu, SP – Brasil

**Keywords:** Poliaminas, Síndrome Metabólica, Doenças Cardiovasculares

## Abstract

**Fundamento:**

As doenças cardiovasculares (DCV) são a principal causa de mortalidade do mundo, e um de seus fatores de risco são os hábitos alimentares não saudáveis, tais como, o alto consumo de frutose. As aminas biogênicas (ABs) realizam funções importantes no corpo humano. Entretanto, o efeito do consumo de frutose nos níveis das ABs ainda não está claro, bem como a associação entre estes e os fatores de risco da DCV.

**Objetivo:**

Este estudo teve o objetivo de estabelecer a associação entre os níveis de ABs e os fatores de risco de DCV em animais que consumiram frutose.

**Métodos:**

Ratos Wistar machos receberam ração convencional (n=8) ou ração convencional + frutose na água de beber (30%) (n=8) durante 24 semanas. Ao final, foram analisados os parâmetros nutricionais e da síndrome metabólica (SM) e os níveis plasmáticos das ABs. Foi adotado um nível de significância de 5%.

**Resultados:**

O consumo de frutose levou à SM, reduziu os níveis de triptofano e 5-hidroxitriptofano e aumentou a histamina. Os níveis de triptofano, histamina e dopamina apresentaram correlação com parâmetros de síndrome metabólica.

**Conclusão:**

O consumo de frutose altera as ABs associadas a fatores de risco de doenças cardiovasculares.

**Figura Central:**
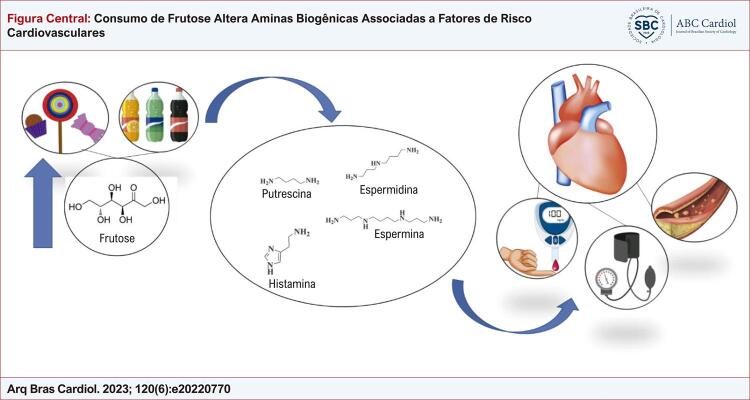
: Consumo de Frutose Altera Aminas Biogênicas Associadas a Fatores de Risco Cardiovasculares

O aumento do consumo de frutose leva a alterações em aminas biogênicas, que estão associadas a fatores de risco cardiovascular, tais como, diabetes tipo 2,hipertensão e dislipidemia.

## Introdução

As doenças cardiovasculares (DCV) são a principal causa de morbidade e mortalidade tanto em países desenvolvidos quanto em desenvolvimento, e apresentam como principais fatores de risco dislipidemia, hipertensão, diabetes, obesidade abdominal, fatores psicossociais, consumo excessivo de álcool, falta de atividade física regular e hábitos alimentares pouco saudáveis.^1^ Um dos principais contribuintes para distúrbios metabólicos associados a DCV é a frutose, um açúcar simples naturalmente presente em frutas, vegetais e mel, que tem sido usado para indústrias alimentícias como adoçante desde a década de 1970. A literatura relata que o consumo crônico e excessivo de produtos industrializados contendo frutose está associado a diversos distúrbios metabólicos^2 - 4^ por mecanismos não totalmente compreendidos.

As aminas biogênicas (ABs) são compostos nitrogenados quimicamente categorizados como monoaminas, diaminas e poliaminas, que podem ser obtidos a partir de alimentos, síntese celular e/ou síntese microbiana no intestino, e estão presentes em todos os organismos vivos, desde bactérias até seres humanos.^5^ Entre todas as ABs, as mais comuns presentes no tipo de célula procariótica e eucariótica são putrescina, espermidina e espermina.^6^ Embora a primeira descrição das ABs remonte ao século XVII, só foram alcançados grandes avanços em seu metabolismo e funções na segunda metade do século passado.^7^

Vários experimentos mostraram que, devido à sua natureza policatiônica, as ABs podem se ligar a biomoléculas carregadas negativamente como DNA, RNA, proteínas e fosfolipídios. Esse comportamento pode modificar os mecanismos celulares de tradução, transcrição, transdução de sinal, proliferação e diferenciação celular, apoptose e resposta celular ao estresse.^8 , 9^ Todas essas modificações foram associadas a alguns distúrbios relacionados à idade, incluindo Alzheimer, câncer e DCV.^5^ Doenças crônicas e relacionadas à idade têm desequilíbrio inflamatório e oxidativo envolvido em sua patogênese, e esses processos podem ser influenciados pelos efeitos anti-inflamatórios e antioxidantes exercidos por algumas ABs.^10^ No entanto, a associação entre ABs e DCV precisa ser elucidada.^7^

A participação do consumo de frutose nas DCV já é demonstrada pela literatura.^2 - 4 , 11 , 12^ Entretanto, o efeito do consumo de frutose nos níveis das ABs ainda é incerto, e sua associação com fatores de risco para DCV precisa ser esclarecida. Assim, este estudo teve como objetivo estabelecer a associação entre níveis de ABs e fatores de risco para DCV em animais que consumiram frutose.

## Materiais e métodos

### Protocolo experimental

Todos os experimentos e procedimentos foram aprovados pelo Comitê de Ética Animal da Faculdade de Medicina de Botucatu (protocolo número 1065/2013) e realizados de acordo com o Guia do Instituto Nacional de Saúde para o Cuidado e Uso de Animais de Laboratório.^13^ Ratos Wistar machos (8 semanas de idade) foram alojados em gaiolas individuais por 24 semanas com condições ambientais controladas (22 °C ± 3 °C; ciclo claro-escuro de 12 horas e umidade relativa de 60 ± 5%). Os animais receberam ração convencional (grupo controle, n=8 animais) ou ração convencional + frutose na água de beber (30%) (grupo frutose, n=8 animais) *ad libitum* . Os animais de cada grupo foram convenientemente alocados de forma a obter o mesmo peso corporal inicial entre os grupos. O tamanho da amostra foi calculado usando SigmaStat para Windows versão 3.5 (Systat Software Inc., San Jose, CA, EUA) considerando as médias de diferença esperadas e desvio padrão esperado para fatores de risco de doença cardiovascular (resistência à insulina, pressão arterial sistólica e dislipidemia), poder de teste de 90% e α de 0,05. Portanto, o tamanho mínimo da amostra é de 6 animais/grupo. Ao final do experimento, foram excluídos deste estudo os animais dos grupos controle que desenvolveram alterações nos fatores de risco para DCV e os animais do grupo frutose que não apresentaram alterações nesses parâmetros. Portanto, cada grupo permaneceu com 8 animais.

### Parâmetros nutricionais

Os parâmetros nutricionais incluíram os seguintes: ingestão calórica, peso corporal final e índice de adiposidade. A ingestão calórica foi determinada pela multiplicação do valor energético da dieta pelo consumo alimentar diário (3,83 kcal x g consumido). A ingestão calórica para o grupo frutose também considerou as calorias provenientes da água (0,30 × 4 × mL consumidos). O peso corporal foi medido semanalmente. Após a eutanásia, os depósitos de gordura visceral (LAV), epididimal (TAE), e retroperitoneal (RAT) foram usados para calcular o índice de adiposidade (IA) pela seguinte fórmula: [(VAT+EAT+RAT)/FBW]×100 .^14 , 15^

### Pressão arterial sistólica

A avaliação da pressão arterial sistólica (PAS) foi realizada em ratos conscientes pelo método não invasivo de manguito de cauda com um eletroesfigmomanômetro NarcoBioSystems® (International Biomedical, Austin, TX, EUA). Os animais foram mantidos em uma caixa de madeira (50 x 40 cm) entre 38–40 °C por 4–5 minutos para estimular a vasodilatação arterial.^16^ Após esse procedimento, um manguito com sensor de pulso pneumático foi fixado à cauda de cada animal. O manguito foi inflado até a pressão de 200 mmHg e, em seguida, esvaziado. Os valores da pressão arterial foram registrados em um polígrafo Gould RS 3200 (Gould Instrumental Valley View, Ohio, EUA). A média de três leituras de pressão foi registrada para cada animal.

### Análise metabólica e hormonal plasmática

Após 12h de jejum, o sangue foi coletado e o plasma foi utilizado para as análises seguintes. Um kit enzimático-colorimétrico foi usado para medir glicose e triglicerídeos (Bioclin®; Belo Horizonte) usando um sistema analisador enzimático automático (Chemistry Analyzer BS-200, Mindray Medical International Limited, Shenzhen, China). O nível de insulina foi medido usando o método de ensaio de imunoabsorção enzimática (ELISA) usando kits comerciais (EMD Millipore Corporation, Billerica, MA, EUA). O modelo homeostático de resistência à insulina (HOMA-IR) foi utilizado como índice de resistência à insulina, calculado de acordo com a fórmula: HOMA-IR = (glicose em jejum (mmol/L) x insulina em jejum (μU/mL))/22,5 .

### Níveis de aminoácidos e ABs por cromatografia líquida de alta eficiência (HPLC) de fase reversa

O plasma (200µL) foi misturado com 800µL de ácido perclórico frio 5% (v/v). Depois disso, a mistura foi centrifugada a 3800 *g* (Hettich Zentrifugen, Mikro220R) por 20 min a 4°C, e ao sobrenadante (200μl) foram adicionados 400μl de cloreto de dansila e 200μl de carbonato de sódio saturado (2M). Após incubação por 1h a 60°C, 200μl de prolina foram adicionados à mistura e mantidos no escuro por 30 min em temperatura ambiente. A essa mistura foram adicionados 1000μl de tolueno mal homogeneizado e o sobrenadante foi seco sob nitrogênio gasoso. As amostras foram ressuspensas em 0,5 mL de acetonitrila e 20µL foram injetados no sistema HPLC (Dionex UltiMate 3000; Thermo Fisher Scientific, Bremen, Alemanha) equipado com bomba quaternária, amostrador automático 3000, detector de arranjo de diodos (DAD-3000) e um ACE C18 coluna (4,6×250mm; 5um) a 25°C. A análise foi monitorada em 280 nm, e o pico de integração e as calibrações foram realizadas entre 210 e 350 nm usando o software Chromeleon 7 (Thermo Fisher Scientific, Bremen, Alemanha). O gradiente do cromatógrafo foi estabelecido para uma mistura de solventes (A) 100% de acetonitrila e (B) 50% de acetonitrila, como segue: 0–2 min, 40% A + 60% B; 2–4 min, 60% A + 40% B; 4–8 min, 65% A + 35% B; 8–12 min, 85% A + 15% B; 12–15 min, 95% A + 5% B; 15–21 min, 85% A + 15% B; 21–22 min, 75% A + 25% B; 22–25 min, 40% A + 60% B. A quantidade de cada AB e aminoácido foi calculada comparando as áreas dos picos com os padrões e a área abaixo da curva obtida por meio de curvas de calibração.

### Análise estatística

Os dados foram submetidos ao teste de normalidade de Kolmogorov-Smirnov. As variáveis paramétricas foram comparadas pelo teste t de Student não pareado e os resultados são relatados como média ± desvio padrão. As variáveis não paramétricas foram comparadas pelo teste de Mann-Whitney e os resultados são relatados como mediana (faixa interquartil (25-75)). A correlação de Spearman foi utilizada para avaliar a associação entre aminas biogênicas e fatores de risco para DCV. As análises estatísticas foram realizadas usando Sigma Stat para Windows versão 3.5 (Systat Software Inc., San Jose, CA, EUA). Um p valor <0,05 foi considerado estatisticamente significativo.

## Resultados

**Figura 1:**
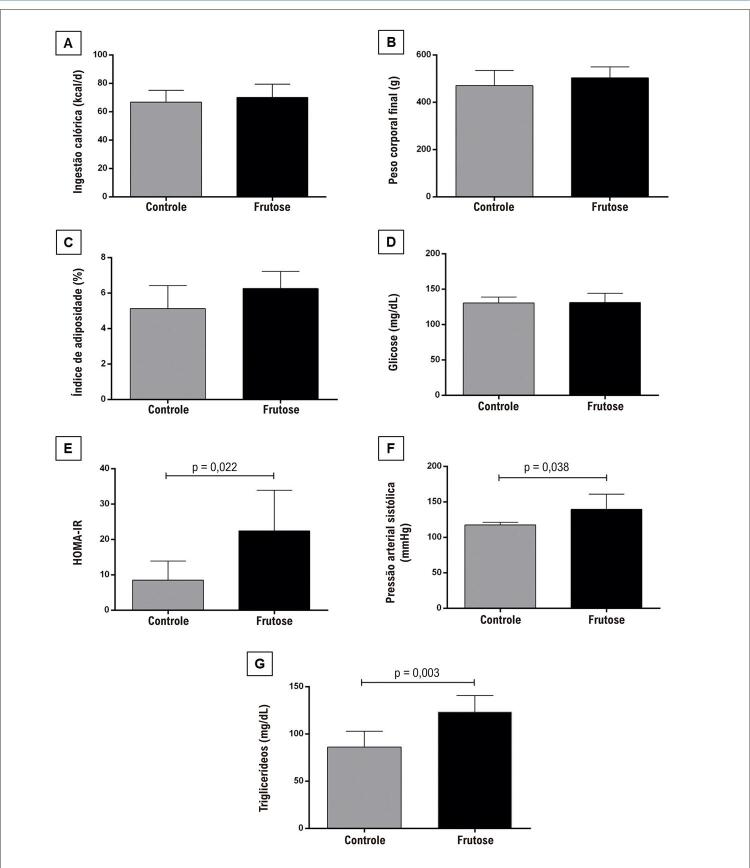
– Fatores de risco para DCV. A) Ingestão calórica (kcal/dia); B) Peso corporal final (g); C) Índice de adiposidade (%); D) Glicose (mg/dl); E) Modelo homeostático de resistência à insulina (HOMA-IR); F) Pressão arterial sistólica (mmHg); G) Níveis de triglicerídeos (mg/dL). Comparação pelo teste T de Student (p<0,05 como significativo).

O peso corporal inicial foi semelhante entre os grupos (grupo controle= 258 ±16 g; grupo frutose= 257 ± 16g, p= 0,80). A figura 1 mostra os parâmetros avaliados em ambos os grupos e associados a maior risco de DCV. É possível notar que o grupo frutose apresentou níveis elevados de pressão arterial sistólica, HOMA-IR e triglicerídeos. Não foram observadas alterações na ingestão calórica, peso corporal final, índice de adiposidade e glicose plasmática.

Os níveis plasmáticos de aminoácidos e ABs são apresentados na tabela 1 . O consumo de frutose reduziu os níveis plasmáticos de triptofano e 5-hidroxitriptofano (5-HTP) e aumentou os níveis de histamina. Não foram observadas alterações para as demais ABs.

**Tabela 1:** – Níveis de aminoácidos e aminas biogênicas

	Controle	Frutose	p valor
Triptofano (µg/mL)	25,5 (24,8, 26,3)	18,4 (16,7, 19,6)	0,003*
5-HTP (µg/mL)	120 (113 - 123)	82,0 (79,0, 88,1)	0,002*
Triptamina (µg/mL)	200 (178 - 245)	192 (166 - 207)	0,996
Agmatina (µg/mL)	3,45 (2,66, 4,52)	3,28 (2,67, 4,04)	1,00
Putrescina (µg/mL)	343 (221 - 343)	257 (215 - 297)	0,310
Histamina (µg/mL)	5,00 (3,84, 6,32)	16,0 (16,0, 17,8)	<0,001*
Tiramina (µg/mL)	17,5 (16,0, 19,4)	21,8 (19,1, 26,3)	0,053
Espermidina (µg/mL)	8,99 (7,75, 11,0)	11,2 (10,5, 11,5)	0,143
Dopamina (µg/mL)	2,30 (1,31, 2,34)	2,70 (2,54, 3,54)	0,132
Espermina (µg/mL)	1,10 (1,04, 1,27)	0,65 (0,59, 0,67)	0,052

Os dados foram expressos como mediana (faixa interquartil). Comparação pelo teste de Mann-Whitney (*p<0,05 considerado significativo). Os valores em negrito representam diferenças estatísticas significativas entre os grupos de frutose e de controle. 5-HTP - 5-hidroxitriptofano

A Tabela 2 apresenta a correlação de Spearman entre os aminoácidos e ABs e os fatores de risco cardiovascular. É possível verificar que os níveis de 5-HTP correlacionaram-se negativamente com insulina, HOMA-IR e triglicerídeos. Os níveis de histamina foram positivamente correlacionados com insulina, HOMA-IR e triglicerídeos. Os níveis de dopamina foram positivamente correlacionados ao HOMA-IR.

**Tabela 2:** – Correlação de Spearman entre as variáveis

	Glicose	Insulina	HOMA	PAS	TG
Triptofano (µg/mL)	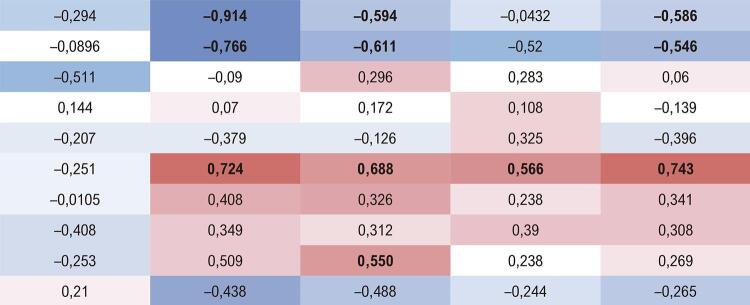					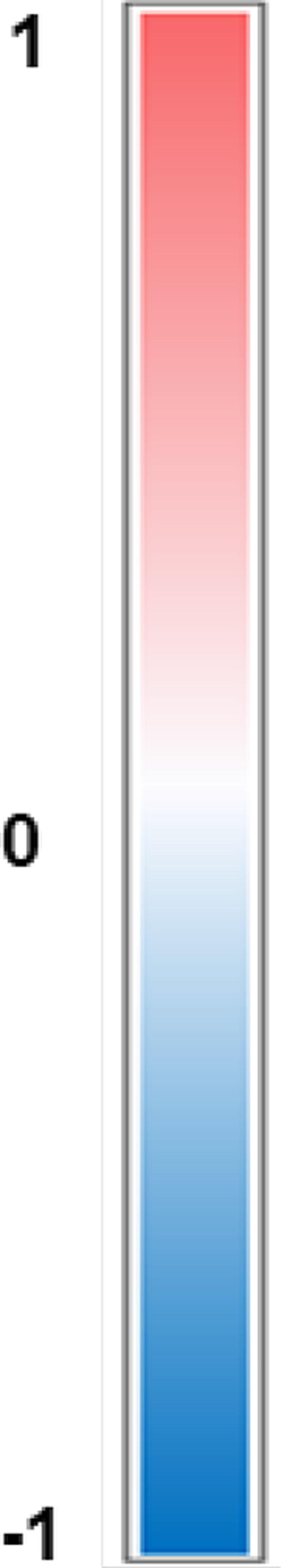				
5-HTP (µg/mL)										
Triptamina (µg/mL)										
Agmatina (µg/mL)										
Putrescina (µg/mL)										
Histamina (µg/mL)										
Tiramina (µg/mL)										
Espermidina (µg/mL)										
Dopamina (µg/mL)										
Espermina (µg/mL)										

PAS: pressão arterial sistólica; TG: triglicerídeos. Valores vermelhos indicam correlação positiva e valores azuis indicam correlação negativa. Quanto mais intensa a cor, maior a correlação. Os valores em negrito mostram os resultados significativos (p<0,05). 5-HTP - 5-hidroxitriptofano.

## Discussão

O objetivo deste estudo foi estabelecer a associação entre ABs e os fatores de risco para DCV em animais que consumiram frutose. A frutose é um açúcar comumente encontrado em frutas. No entanto, a indústria tem utilizado o xarope de milho, rico em frutose, como adoçante para bebidas e alimentos, aumentando a ingestão desse açúcar pela população.^3^ O alto consumo de frutose pode levar ao aumento da obesidade e comorbidades relacionadas à obesidade, como dislipidemia, resistência à insulina, hipertensão e diabetes mellitus tipo II, todos fatores de risco para DCV.^17 , 18^ Dentro desse contexto, é possível verificar que a ingestão de frutose foi capaz de induzir dislipidemia, resistência à insulina e hipertensão nos animais, confirmando os efeitos negativos de seu consumo. Além disso, o grupo frutose apresentou diminuição dos níveis plasmáticos de triptofano e 5HTP e aumento dos níveis de histamina.

O triptofano é um aminoácido essencial para o ser humano envolvido em vias metabólicas cruciais que resultam em vários produtos finais, entre os quais as proteínas. O triptofano tem sido implicado em várias doenças e condições, incluindo DCV, devido ao seu papel fundamental como precursor de muitos metabólitos bioativos. Também foi demonstrado que o triptofano suprime os níveis séricos de glicose e insulina e inibe a absorção de glicose no intestino, sugerindo que esse aminoácido suprime a elevação dos níveis de glicose no sangue e reduz os efeitos adversos associados à insulina plasmática elevada.^19^ Algumas evidências indicam que o triptofano serve como fonte de nitrogênio para o crescimento de alguns microrganismos patogênicos no intestino, o que representa uma perturbação da microbiota intestinal. Essa condição também pode explicar os níveis reduzidos de triptofano no grupo da frutose. A disbiose intestinal tem sido associada a DCV, especialmente por alterar o metabolismo da glicose e lipídios e desencadear inflamação.^20^

O 5-HTP é produzido a partir do triptofano pela triptofano hidroxilase (TPH) e sua descarboxilação produz a serotonina (5-hidroxitriptamina, 5-HT), um neurotransmissor monoamínico envolvido na modulação do humor, cognição, recompensa, aprendizado, memória, sono e vários outros processos fisiológicos.^21^ A serotonina intestinal tem diversas funções em sistemas neuronais e não neuronais, atuando como hormônio e mitógeno, além de neurotransmissor. A serotonina regula diversos processos fisiológicos e patológicos, que são mediados por inúmeros receptores, destacando-se o 5-HT2B que se expressa em tecidos periféricos como fígado, rim, coração e estômago. Está associada à função cardíaca, à doença cardíaca valvular, e à morfogênese cardíaca.^22^ Entretanto, embora muitas funções do 5-HPT ocorram nos tecidos, mais de 90% do 5-HTP corporal total são produzidos no intestino. A serotonina é liberada por células e neurônios enterocromafins e é regulada por meio do transportador de recaptação de serotonina (SERT), que está localizado nas células epiteliais e nos neurônios do intestino.^23^ No entanto, a literatura já relatou que alguns nutrientes, como a frutose, promovem danos à barreira intestinal e comprometimento do SERT, levando à translocação de lipopolissacarídeos (LPS). O comprometimento do SERT pode explicar os níveis mais baixos de 5-HTP nos animais que consumiram frutose. Além disso, a translocação do LPS está associada à inflamação, condição que explica as alterações metabólicas apresentadas pelo grupo frutose mesmo na ausência de obesidade.^24 , 25^ Outra explicação para níveis mais baixos de 5-HTP em baixos níveis de triptofano, uma vez que este aminoácido é substrato para sintetizar 5-HTP.^26^

A histamina é uma amina de baixo peso molecular sintetizada a partir da L-histidina exclusivamente pela histidina descarboxilase e produzida por várias células em todo o corpo. Participa da regulação de muitas funções fisiológicas, incluindo proliferação e diferenciação celular, hematopoiese, desenvolvimento embrionário e regeneração. No sistema nervoso central, afeta a cognição e a memória, a regulação do ciclo do sono e da vigília, a homeostase energética e endócrina. A histamina é um neurotransmissor bem conhecido envolvido em condições alérgicas e fisiológicas que podem regular as funções cardiovasculares.^27^ Os níveis plasmáticos aumentados de histamina foram significativamente correlacionados com os fatores de risco de doença cardíaca observados no grupo da frutose. No entanto, faltam estudos que avaliem os níveis de ABs em indivíduos com comprometimento metabólico decorrente da ingestão de frutose. Outras ABs analisadas, como a putrescina e a tiramina, embora não tenham apresentado correlação com fatores de risco cardíaco, poderiam estar potencializando a toxicidade da histamina ao interagir com as aminas oxidases e favorecer a absorção intestinal e diminuir a desintoxicação da histamina.^7^ No entanto, o grupo frutose apresentou menores teores de triptofano, o que pode ser explicado pela reação que pode ocorrer entre a frutose e proteínas e aminoácidos como o triptofano, levando à formação de um complexo frutose-triptofano que pode resultar na diminuição da qualidade da proteína devido à perda de resíduos de aminoácidos e diminuição da digestibilidade da proteína.^28^

Embora evidências substanciais tenham se acumulado sobre o metabolismo, receptores e transdução de sinal da histamina, a complexa inter-relação e interferência da histamina e os efeitos fisiológicos e patológicos precisam ser esclarecidos. Em humanos, a histamina desencadeia sintomas agudos devido à sua atividade muito rápida no endotélio vascular e nas células brônquicas e musculares lisas, levando a sintomas como rinite aguda, broncoconstrição, cólicas, diarreia ou vermelhidão cutânea e respostas exacerbadas.^29 , 30^ Apesar da alta variabilidade dos teores de ABs nos alimentos, a dieta ocidental moderna é caracterizada por quantidades excessivas de frutose. Essas grandes quantidades sobrecarregam o transportador de frutose nas células epiteliais intestinais, resultando em maiores quantidades de frutose não completamente absorvidas pela mucosa intestinal ou ainda, intolerância à frutose.^31^ A intolerância à frutose é uma das condições associadas a reações adversas à histamina, também conhecida como intolerância à histamina. É uma condição causada por um desequilíbrio entre a histamina liberada dos alimentos e a capacidade do organismo de degradar tal quantidade, levando a um aumento da concentração de histamina no plasma e ao surgimento de reações adversas.^32 , 33^ Essa condição de intolerância à frutose pode explicar os altos níveis de histamina encontrados no plasma de animais que consumiram frutose.^34^

### Limitações do estudo

A limitação deste estudo é não avaliar as vias pelas quais as aminas biogênicas estão associadas a DCV.

## Conclusão

Em conclusão, este estudo mostrou que o consumo de frutose leva à resistência à insulina, aumento da pressão arterial sistólica e dislipidemia. Também foi demonstrado que a ingestão de frutose alterou os níveis de aminas biogênicas, principalmente por aumentar a histamina e reduzir os níveis de triptofano e 5-hidroxitriptofano, e essas alterações podem estar associadas a fatores de risco para DCV.
