# Supplementary material for: Consumo de Frutose Altera Aminas Biogênicas Associadas a Fatores de Risco Cardiovasculares
Source: Arq Bras Cardiol. 2023 Jun 7;120(6):e20220770. [Article in Portuguese] doi: 10.36660/abc.20220770 (PMC10263407; doi:10.36660/abc.20220770)
Supplement: Translation

# Translation: Fructose Consumption Alters Biogenic Amines Associated with Cardiovascular Disease Risk Factors

**Authors:** Fabiane Valentini Francisqueti-Ferron, Matheus Antônio Filiol Belin, Thiago Luiz Novaga Palacio, Artur Junio Togneri Ferron, Jéssica Leite Garcia, Juliana Silva Siqueira, Erika Tiemi Nakandakare-Maia, Taynara Aparecida Vieira, Hugo Tadashi Kano, Fernando Moreto, Giuseppina Pace Pereira Lima, Camila Renata Corrêa, Igor Otavio Minatel

**Affiliations:** 1 Brazil Universidade Estadual Paulista Júlio de Mesquita Filho Câmpus de Botucatu Faculdade de Medicina, Botucatu, SP – Brazil

**Keywords:** Polyamines, Metabolic Syndrome, Cardiovascular Diseases

## Abstract

**Background:**

Cardiovascular diseases (CVD) are the major cause of mortality worldwide, whose most prominent risk factor is unhealthy eating habits, such as high fructose intake. Biogenic amines (BAs) perform important functions in the human body. However, the effect of fructose consumption on BA levels is still unclear, as is the association between these and CVD risk factors.

**Objective:**

This study aimed to establish the association between BA levels and CVD risk factors in animals that consumed fructose.

**Methods:**

Male Wistar rats received standard chow (n=8) or standard chow + fructose in drinking water (30%) (n=8) over a 24-week period. At the end of this period, the nutritional and metabolic syndrome (MS) parameters and plasmatic BA levels were analyzed. A 5% level of significance was adopted.

**Results:**

Fructose consumption led to MS, reduced the levels of tryptophan and 5-hydroxitryptophan, and increased histamine. Tryptophan, histamine, and dopamine showed a correlation with metabolic syndrome parameters.

**Conclusion:**

Fructose consumption alters BAs associated with CVD risk factors.

**Central Illustration:** : Fructose Consumption Alters Biogenic Amines Associated with Cardiovascular Disease Risk Factors

Increased fructose intake leads to changes in biogenic amines, which are associated with cardiovascular risk factors, such as type 2 Diabetes, hypertension, and dyslipidemia.

## Introduction

Cardiovascular diseases (CVD) are the major cause of morbidity and mortality in both developed and developing countries, presenting the main risk factors of dyslipidemia, hypertension, diabetes, abdominal obesity, psychosocial factors, excessive alcohol consumption, the lack of regular physical activity, and unhealthy eating habits.^1^ One of the major contributors to metabolic disorders associated with cardiovascular disease is fructose, a simple sugar naturally present in fruits, vegetables, and honey, which has been used for food industries as a sweetener since the 1970s. The literature reports that the chronic and excessive consumption of industrialized products containing fructose is associated with several metabolic disorders^2 - 4^ by mechanisms that are not fully understood.

Biogenic amines (BAs) are nitrogenous compounds chemically categorized as monoamines, diamines, and polyamines, which can be obtained from food, cell synthesis, and/or microbial synthesis in the gut, and are present in all living organisms from bacteria to humans.^5^ Among all BAs, the most common types, which appear in prokaryotic and eukaryotic cell types, are putrescine, spermidine, and spermine.^6^ Although the first description of BAs dates back to the seventeenth century, major advances in their metabolism and functions were achieved only in the second part of the last century.^7^

Several experiments have shown that, due to their polycationic nature, BAs can bind into negatively charged biomolecules, such as DNA, RNA, proteins, and phospholipids. This behavior can modify a cell’s mechanism of translation, transcription, signal transduction, cell proliferation and differentiation, apoptosis, and cell stress response.^8 , 9^ All these modifications have been associated with some age-related disorders, including Alzheimer, cancer, and CVD.^5^ Chronic and age-related diseases have an inflammatory and oxidative imbalance involved in their pathogenesis, and these processes may be influenced by the anti-inflammatory and antioxidant effects exerted for some BAs.^10^ However, the association between BAs and CVDs needs to be elucidated.^7^

The participation of fructose consumption in cardiovascular diseases has already been demonstrated by the literature.^2 - 4 , 11 , 12^ However, the effect of fructose consumption on BA levels is still lacking, and its association with CVD risk factors needs to be clarified. Thus, this study aimed was to establish the association between BA levels and CVD risk factors in animals that consumed fructose.

## Material and methods

### Experimental Protocol

All the experiments and procedures were approved by the Animal Ethics Committee of Botucatu Medical School (protocol number 1065/2013) and were performed in accordance with the National Institute of Health’s Guide for the Care and Use of Laboratory Animals.^13^ Male Wistar rats (8 weeks old) were housed in individual cages for 24 weeks under environmentally controlled conditions (22 °C ± 3 °C; 12-h light-dark cycle and relative humidity of 60 ± 5%). Animals received standard chow (control group, n=8 animals) or standard chow + fructose in drinking water (30%) (fructose group, n=8 animals) *ad libitum* . Animals of each group were conveniently allocated in order to obtain the same initial body weight between the groups. The sample size was calculated by using SigmaStat for Windows Version 3.5 (Systat Software Inc., San Jose, CA, USA) considering the means of expected difference and expected standard deviation for CVD risk factors (insulin resistance, systolic blood pressure, and dyslipidemia), a test power of 90%, and α of 0.05. Therefore, the minimum sample size was 6 animals/ group. At the end of the experiment, animals from control groups that developed changes in CVD risk factors and animals from the fructose group that did not show changes in these parameters were excluded from this study. Thus, each group remained with 8 animals.

### Nutritional Parameters

The nutritional parameters included the following parameters: caloric intake, final body weight, and adiposity index. Caloric intake was determined by multiplying the energy value of diet by the daily food consumption (3.83 kcal x g consumed). The caloric intake for the fructose group also considered the calories from water (0.30 × 4 × mL consumed). Body weight was measured weekly. After euthanasia, the visceral (VAT), epididymal (EAT), and retroperitoneal (RAT) fat deposits were used to calculate the adiposity index (AI) by the following formula: [(VAT+EAT+RAT)/FBW]×100 .^14 , 15^

### Systolic blood pressure

The evaluation of systolic blood pressure (SBP) was assessed in conscious rats by the non-invasive tail-cuff method with a NarcoBioSystems^®^Electro-Sphygmomanometer (International Biomedical, Austin, TX, USA). The animals were kept in a wooden box (50 x 40 cm) between 38–40°C for 4–5 minutes to stimulate arterial vasodilation.^16^ After this procedure, a cuff with a pneumatic pulse sensor was attached to the tail of each animal. The cuff was inflated to 200 mmHg pressure and subsequently deflated. The blood pressure values were recorded on a Gould RS 3200 polygraph (Gould Instrumental Valley View, Ohio, USA). The average of three pressure readings was recorded for each animal.

### Plasma metabolic and hormonal analysis

After 12h of fasting, blood was collected and plasma was used for the following analysis. An enzymatic- colorimetric kit was used to measure glucose and triglycerides (Bioclin^®^; Belo Horizonte) using an automatic enzymatic analyzer system (Chemistry Analyzer BS-200, Mindray Medical International Limited, Shenzhen, China). The insulin level was measured using the enzyme-linked immunosorbent assay (ELISA) method using commercial kits (EMD Millipore Corporation, Billerica, MA, USA). The homeostatic model of insulin resistance (HOMA-IR) was used as an insulin resistance index, calculated according to the formula: HOMA-IR = (fasting glucose (mmol/L) x fasting insulin (μU/mL))/22.5 .

### Amino acids and BAs levels by reverse-phase high-performance liquid chromatography (HPLC)

The plasma (200µL) was mixed with 800µL of cold perchloric acid 5% (v/v). The mixture was then centrifuged at 3800 *g* (Hettich Zentrifugen, Mikro220R) for 20 min at 4°C, and 400μl of dansyl chloride and 200μl of saturated sodium carbonate (2M) were added to the supernatant (200 μl). After incubation for 1 h at 60°C, 200μl of proline was added to the mixture and maintained in the dark for 30 min at room temperature. To this mixture was added 1000μl of toluene, strongly homogenated, and the supernatant was dried under gaseous nitrogen. The samples were resuspended in 0.5mL of acetonitrile and 20µL were injected into the HPLC system (Dionex UltiMate 3000; Thermo Fisher Scientific, Bremen, Germany) equipped with quaternary pump, automatic sampler 3000, diode array detector (DAD-3000), and an ACE C18 column (4.6 × 250 mm; 5um) at 25°C. The analysis was monitored at 280nm, and the integration peak and calibrations were realized between 210 and 350nm using Chromeleon 7 software (Thermo Fisher Scientific, Bremen, Germany). The chromatograph gradient was set to a mixture of solvents, (A) 100% acetonitrile and (B) 50% acetonitrile, as follows: 0–2 min, 40% A + 60% B; 2–4 min, 60% A + 40% B; 4–8 min, 65% A + 35% B; 8–12 min, 85% A + 15% B; 12–15 min, 95% A + 5% B; 15–21 min, 85% A + 15% B; 21–22 min, 75% A + 25% B; 22–25 min, 40% A + 60% B. The amount of each BA and amino acid was calculated by comparing the peak areas with standards and the area under the curve (AUC) obtained through calibration curves.

### Statistical analysis

The data were submitted to the Kolmogorov-Smirnov normality test. Parametric variables were compared by unpaired Student’s t-test, and the results are reported as mean ± standard deviation. Non-parametric variables were compared using the Mann-Whitney test, and the results were reported as median (interquartile range (25-75)). Spearman correlation was used to assess the association among biogenic amines and CVD risk factors. Statistical analyses were performed using Sigma Stat for Windows Version 3.5 (Systat Software Inc., San Jose, CA, USA). A p value < 0.05 was considered statistically significant.

## Results

Initial body was similar between the groups (control group= 258 ±16 g; fructose group= 257 ± 16g, p= 0.80). Figure 1 shows parameters assessed in both groups and associated with a higher risk for CVD. It is possible to note that the fructose group presented high systolic blood pressure, HOMA-IR, and triglyceride levels. No changes were observed in caloric intake, final body weight, adiposity index, and plasmatic glucose.

**Figure 1:** – Cardiovascular disease (CVD) risk factors. A) Caloric intake (kcal/day); B) Final body weight (g); C) Adiposity index (%); D) Glucose (mg/dl); E) Homeostatic model of insulin resistance (HOMA-IR); F) Systolic blood pressure (mmHg); G) Triglyceride levels (mg/dL). Comparison conducted using the Student’s T test (p<0.05 considered significant).

Plasmatic levels of amino acids and BAs are presented in the Table 1 . The fructose consumption reduced the plasma levels of tryptophan and 5-hydroxytryptophan (5-HTP) and increased the levels of histamine. No changes were observed for the other BAs.

**Table 1:** – Plasma amino acids and biogenic amines levels

	Control	Fructose	p value
Tryptophan (µg/mL)	25.5 (24.8 – 26.3)	18.4 (16.7 - 19.6)	0.003*
5-HTP (µg/mL)	120 (113 - 123)	82.0 (79.0 - 88.1)	0.002*
Tryptamine (µg/mL)	200 (178 - 245)	192 (166 - 207)	0.996
Agmatine (µg/mL)	3.45 (2.66 - 4.52)	3.28 (2.67 - 4.04)	1.00
Putrescine (µg/mL)	343 (221 - 343)	257 (215 - 297)	0.310
Histamine (µg/mL)	5.00 (3.84 - 6.32)	16.0 (16.0 - 17.8)	< 0.001*
Tyramine (µg/mL)	17.5 (16.0 - 19.4)	21.8 (19.1 - 26.3)	0.053
Spermidine (µg/mL)	8.99 (7.75 - 11.0)	11.2 (10.5 - 11.5)	0.143
Dopamine (µg/mL)	2.30 (1.31 - 2.34)	2.70 (2.54 - 3.54)	0.132
Spermine (µg/mL)	1.10 (1.04 - 1.27)	0.65 (0.59 - 0.67)	0.052

Data are presented as median (interquartile range). Comparison conducted using the Mann-Whitney test (*p<0.05 as significant). Bold values represent significative statistical differences between fructose and control groups. 5-HTP - 5-hydroxytryptophan.

Table 2 presents the Spearman Correlation among the amino acids and BAs and CVD risk factors. It is possible to verify that the levels of 5-HTP were negatively correlated with insulin, HOMA-IR and triglycerides. Histamine levels were positively correlated with insulin, HOMA-IR, and triglycerides. Dopamine levels were positively correlated with HOMA-IR.

**Table 2:** – Spearman correlation among the variables

	Glucose	Insulin	HOMA	SBP	TG
Tryptophan (µg/mL)										
5-HTP (µg/mL)										
Tryptamine (µg/mL)										
Agmatine (µg/mL)										
Putrescine (µg/mL)										
Histamine (µg/mL)										
Tyramine (µg/mL)										
Spermidine (µg/mL)										
Dopamine (µg/mL)										
Spermine (µg/mL)										

SBP: systolic blood pressure; TG: triglycerides. Red values indicate positive correlation and blue values indicate negative correlation. The more intense the color, the greater the correlation. Bold values show the significant results (p<0.05). 5-HTP - 5-hydroxytryptophan.

## Discussion

The aim of this study was to establish the association between BAs and CVD risk factors in animals that consumed fructose. Fructose is a sugar commonly found in fruits. However, the industry has used corn syrup, which is rich in fructose, as a sweetener for beverages and foods, increasing the intake of this sugar by the population.^3^ High fructose consumption can lead to increased obesity and obesity-related comorbidities, such as dyslipidemia, insulin resistance, hypertension, and diabetes mellitus type II, all risk factors for CVD.^17 , 18^ Within this context, it is possible to verify that the fructose intake was able to induce dyslipidemia, insulin resistance, and hypertension in the animals, confirming the negative effects of its consumption. In addition, the fructose group showed decreased plasmatic levels of tryptophan and 5HTP, as well as increased levels of histamine.

Tryptophan is an essential amino acid for humans involved in crucial metabolic pathways that results in various end-products, among them proteins. Tryptophan has been found in various diseases and conditions, including CVD, because of its key role as a precursor of many bioactive metabolites. It was also demonstrated that tryptophan suppresses both serum glucose and insulin levels and inhibits glucose absorption from the intestine, suggesting that this amino acid suppresses the elevation of blood glucose levels and reduces the adverse effects associated with high plasm insulin.^19^ Some evidence has indicated that tryptophan serves as a nitrogen source for the growth of some pathogenic microbes in the gut, which represents an intestinal microbiota perturbation. This condition may also explain the reduced tryptophan levels in the fructose group. Gut dysbiosis has been associated with CVD, especially by altering glucose and lipid metabolism and triggering inflammation.^20^

5-HTP is produced from tryptophan by tryptophan hydroxylase (TPH), and its decarboxylation yields serotonin (5-hydroxytryptamine, 5-HT), a monoamine neurotransmitter involved in the modulation of mood, cognition, reward, learning, memory, sleep, and numerous other physiological processes.^21^ Intestinal serotonin has diverse functions in neuronal and non-neuronal systems, by acting as a hormone and a mitogen, as well as a neurotransmitter. Serotonin regulates several physiological and pathological processes, which are mediated through numerous receptors, highlighting the 5- HT2B expressed in peripheral tissues including the liver, kidney, heart, and stomach. It is associated with cardiac function, valvular heart disease, and heart morphogenesis.^22^ However, although many functions of 5-HTP occur in tissues, over 90% of the total body 5-HTP is produced in the gut. Serotonin is released by enterochromaffin cells and neurons, and is regulated via the serotonin re-uptake transporter (SERT), which is located on epithelial cells and neurons in the gut.^23^ However, the literature has already reported that some nutrients, such as fructose, promote damage to intestinal barriers and an impairment of SERT, leading to lipopolysaccharide (LPS) translocation. The impairment of SERT can explain the lower levels of 5-HTP in the animals that consumed fructose. Moreover, LPS translocation is associated with inflammation, a condition that explains the metabolic changes presented by the fructose group even in the absence of obesity,^24 , 25^ which is another explanation for lower 5-HTP levels in low levels of tryptophan, since this amino acid is a substrate used to synthesize 5-HTP.^26^

Histamine is a low-molecular-weight amine synthesized from L-histidine exclusively by histidine decarboxylase and produced by various cells throughout the body. It participates in the regulation of many physiological functions, including cell proliferation and differentiation, hematopoiesis, embryonic development, and regeneration. In the central nervous system, it affects cognition and memory, the regulation of the cycles of sleeping and waking, as well as energy and endocrine homeostasis. Histamine is a well-known neurotransmitter involved in allergic and physiological conditions that can regulate cardiovascular functions.^27^ The increased plasm levels of histamine were significantly correlated with CVD risk factors observed in the fructose group. However, there is a lack of studies evaluating BA levels in individuals with metabolic impairment arising from fructose intake. Other analyzed BAs, such as putrescine and tyramine, although these have presented no correlation with cardiac risk factors, could be enhancing the histamine toxicity by interacting with amine oxidases, thus favoring intestinal absorption and decreasing histamine detoxification.^7^ However, the fructose group presented lower levels of tryptophan, which can be explained by the reaction that can occurs between fructose and proteins and amino acids, such as tryptophan, leading to a fructose-tryptophan complex that can be formed, which results in a decrease in protein quality due to the loss of amino acid residues and decreased protein digestibility.^28^

Although substantial evidence has accumulated on histamine metabolism, receptors, and signal transduction, the complex interrelationship and cross-talk by histamine and physiological and pathological effects needs to be clarified. In humans, histamine triggers acute symptoms due to its very rapid activity on vascular endothelium and bronchial and smooth muscle cells, leading to such symptoms as acute rhinitis, bronchoconstriction, cramping, diarrhea, or cutaneous weal and flare responses.^29 , 30^ Despite the high variability of the BA content in foods, the modern Western diet is characterized by excessive amounts of fructose. These large quantities overload the fructose transporter on the intestinal epithelial cells, resulting in larger amounts of fructose not completely absorbed by the intestinal mucosa, or even fructose intolerance.^31^ Fructose intolerance is one of the conditions associated with adverse reactions to histamine, also known as histamine intolerance. It is a condition caused by an imbalance between the histamine released from food and the ability of the organism to degrade such an amount, leading to an increased concentration of histamine in plasma and the emergence of adverse reactions.^32 , 33^ This condition of fructose intolerance can explain the high levels of histamine found in the plasma of animals that consumed fructose.^34^

### Study limitations

The limitation of this study is not evaluating the pathways by which biogenic amines are associated with cardiovascular diseases.

## Conclusion

In conclusion, this study showed that fructose consumption led to insulin resistance, increased systolic blood pressure and dyslipidemia. It was also demonstrated that the fructose intake altered biogenic amines levels, mainly by increasing histamine and reducing tryptophan and 5-hydroxytryptophan levels, and these changes may be associated with CVD risk factors.
